# The two phyto-oestrogens genistein and quercetin exert different effects on oestrogen receptor function

**DOI:** 10.1038/sj.bjc.6690479

**Published:** 1999-06

**Authors:** P Miodini, L Fioravanti, G Di Fronzo, V Cappelletti

**Affiliations:** Istituto Nazionale per lo Studio e la Cura dei Tumori, Milan, Italy; CNR Center for Research in Cell Pathology, CNR, Milan, Italy

**Keywords:** genistein, quercetin, oestrogen receptor, breast cancer, phyto-oestrogens

## Abstract

We compared the oestrogenic and anti-oestrogenic properties of the two well-known phyto-oestrogens, genistein and quercetin, on the oestrogen-sensitive breast cancer cell line MCF-7. Genistein exerted a biphasic effect on growth of MCF-7 cells, stimulating at low and inhibiting at high concentrations, whereas quercetin was only growth inhibitory. At doses which did not inhibit cell growth, respectively 5 and 1 μM, genistein and quercetin counteracted oestrogen- and transforming growth factor-α-promoted cell growth stimulation. Furthermore, genistein promoted transcription of the oestrogen-regulated genes pS2 and cathepsin-D, whereas quercetin interfered with the oestrogen-induced expression of the proteins. In in vitro binding experiments, genistein competed with oestradiol for binding to the oestrogen receptor (ER), but quercetin did not. Quercetin and genistein down-regulated cytoplasmic ER levels and promoted a tighter nuclear association of the ER, but only genistein was able to up-regulate progesterone receptor protein levels. In gel mobility assays, ER preincubation with oestradiol or with the two phyto-oestrogens led to the appearance of the same retarded band, excluding differences between the various complexes in binding to the consensus sequence. The data allowed us to conclude that quercetin acts like a pure anti-oestrogen, whereas genistein displays mixed agonist/antagonist properties, and to formulate a hypothesis on the possible mechanism of action of such phyto-oestrogens. © 1999 Cancer Research Campaign


British Journal of Cancer (1999) **80**(8), 1150–1155
© 1999 Cancer Research Campaign
Article no. bjoc.1999.0479

### The two phyto-oestrogens genistein and quercetin exert different effects on oestrogen receptor function

**P Miodini[1], L Fioravanti[1], G Di Fronzo[1,2] and V Cappelletti[1]**

[1]Istituto Nazionale per lo Studio e la Cura dei Tumori, Milan, Italy; [2]CNR Center for Research in Cell Pathology, CNR, Milan, Italy

**Summary** We compared the oestrogenic and anti-oestrogenic properties of the two well-known phyto-oestrogens, genistein and quercetin, on the oestrogen-sensitive breast cancer cell line MCF-7. Genistein exerted a biphasic effect on growth of MCF-7 cells, stimulating at low and inhibiting at high concentrations, whereas quercetin was only growth inhibitory. At doses which did not inhibit cell growth, respectively 5 and 1 μM, genistein and quercetin counteracted oestrogen- and transforming growth factor-α-promoted cell growth stimulation. Furthermore, genistein promoted transcription of the oestrogen-regulated genes pS2 and cathepsin-D, whereas quercetin interfered with the oestrogen-induced expression of the proteins. In in vitro binding experiments, genistein competed with oestradiol for binding to the oestrogen receptor (ER), but quercetin did not. Quercetin and genistein down-regulated cytoplasmic ER levels and promoted a tighter nuclear association of the ER, but only genistein was able to up-regulate progesterone receptor protein levels. In gel mobility assays, ER preincubation with oestradiol or with the two phyto-oestrogens led to the appearance of the same retarded band, excluding differences between the various complexes in binding to the consensus sequence. The data allowed us to conclude that quercetin acts like a pure anti-oestrogen, whereas genistein displays mixed agonist/antagonist properties, and to formulate a hypothesis on the possible mechanism of action of such phyto-oestrogens.

**Keywords:** genistein; quercetin; oestrogen receptor; breast cancer; phyto-oestrogens

The two phyto-oestrogens genistein and quercetin have been reported to play a role in diet-related cancer risk and have recently attracted research interest for their potential chemopreventive activity (Setchell and Adlercreutz, 1988; Adlercreutz, 1990).

Genistein, but not quercetin, is structurally similar to 17β-oestradiol. A number of studies (Lock, 1991; Adlercreutz et al, 1995) have proposed that the low incidence of breast cancer and the mild menopause-related symptoms observed in Asian women (Ross et al, 1991) may be linked to the weak oestrogenic activity of genistein, which is prevalently contained in soy beans and its derivatives. Genistein has also received particular attention due to its oestrogenic and antiproliferative properties in animal models (Barnes et al, 1990; Lamartiniere et al, 1995) as well as in vitro models (Yamagihara et al, 1993; Barnes, 1995; Zava and Duwe, 1997).

Quercetin is contained in most edible fruits and vegetables (Kühnau, 1976) and has been shown to exert growth inhibitory activity on human breast (Scambia et al, 1993; Singhal et al, 1995), ovarian (Scambia et al, 1990), leukaemic (Larocca et al, 1990) and colon (Shiu-Ming Kuo, 1996) cancer cells.

Many different mechanisms of action have been proposed to explain the growth-inhibitory activity of such phyto-oestrogens, such as direct inhibition of tyrosine kinase activity (Akiyama et al, 1987), interaction with oestrogen receptor (ER) or with type-II oestrogen binding sites (Martin et al, 1978), and inhibition of DNA-topoisomerase II (Markovits et al, 1989). Most of the proposed cellular targets for phyto-oestrogens are directly or indirectly related to cell proliferation and may explain the growth-inhibitory effects of such molecules, as well as the cell growth-stimulatory activity presumably linked to the oestrogenic properties of phyto-oestrogens. However, the exact mechanism underlying the in vivo antiproliferative effect as well as the growth-stimulatory activity, which has been observed only in ER-positive cell lines (Fioravanti et al, 1998), has not yet been clarified.

In the present study, we compared in a typically oestrogen-sensitive breast cancer cell line (MCF-7) the effects exerted by genistein and by quercetin on oestrogen-mediated pathways in order to gain insight into their mechanism at the molecular level. The first part of the study addressed the effects of genistein and quercetin on: (1) basal and stimulated growth of MCF-7 cells; (2) steroid receptor modulation; and (3) expression of oestrogen-regulated genes. In the second part, we focused on the molecular mechanism underlying the different biocharacters (i.e. agonist/antagonist) of the two phyto-oestrogens.

### MATERIALS AND METHODS

### Cell lines

MCF-7 cells (kindly provided by K Horwitz, University of Colorado at Denver) were routinely maintained in DMEM/F12 (Sigma) without phenol red and supplemented with 2% fetal calf serum (FCS) and 4 g $l^{-1}$ glucose.

### Cell growth experiments

Experiments were run in 24-well plates or in T-75 or T-150 flasks, in DMEM/F12 supplemented with 2% FCS, in serum-free $MOM_3$ medium (Cappelletti et al, 1993), or in DMEM/F12 supplemented with 2% dextran-coated charcoal-stripped FCS (DCC-FCS) as described by Soto and Sonnenschein (1985). Cell growth was determined by total cell DNA evaluated directly in the 24 wells

*Received 11 August 1998*
*Revised 12 January 1999*
*Accepted 12 January 1999*

*Correspondence to:* V Cappelletti, Oncologia Sperimentale C, Istituto Nazionale Tumori, via Venezian 1, I-20133, Italy

**Table 1** Effect of quercetin and genistein on the expression of steroid receptors

| | ER (fmol mgP$^{-1}$) | PgR (fmol mgP$^{-1}$) |
|---|---|---|
| Control | 251[a] | 6 |
| $E_2$ $10^{-8}$ M | 6 | 252 |
| Quercetin 2.5 μM | 245 | 9 |
| Genistein 5 μM | 118 | 56 |
| Quercetin + $E_2$ | 9 | 81 |
| Genistein + $E_2$ | 13 | 339 |

[a]fmol mgP$^{-1}$ representing the mean of three separate receptor determinations. Each experimental point was run in triplicate in parallel. Standard error among the triplicates was always less than 10%.

**Table 2** MCF-7 cytoplasmic and nuclear ER content after a 6-day treatment with phyto-oestrogens

| | Cytoplasmic ER (fmol mgP$^{-1}$) | Nuclear ER (fmol mgP$^{-1}$) |
|---|---|---|
| Control | 190[a] | 280 |
| 1 μM quercetin | 121 | 335 |
| 5 μM genistein | 104 | 670 |

[a]fmol mgP$^{-1}$ representing the mean of three separate receptor determinations. Each experimental point was performed in parallel in triplicate. Standard error of triplicates was always less than 10%.

with the diphenylamine assay (Burton, 1956). Linearity between cell number variations and DNA content of the wells was checked.

### Steroid receptor determination

Cells ($1 \times 10^8$), harvested by trypsinization, were homogenized in 20 mM $K_2HPO_4$, 1 mM EDTA, 10% glycerol and 12 mM thioglycerol, pH 7.4, with a Potter Teflon/glass homogenizer, and centrifuged to obtain crude cytosol and nuclei. Cytosolic ER and progesterone receptors (PR) were simultaneously estimated by a double-labelling DCC assay as described (Ronchi et al, 1986). Nuclear pellets were salt-extracted as described (Cappelletti et al, 1988), and cytosol and nucleosol were incubated overnight with 16α-[$^{125}$I]-iodo-oestradiol (8150 GBq mmol$^{-1}$, 5 nM), alone or in the presence of a 200-fold molar excess of oestradiol. Incubation was stopped by treatment with a DCC pellet.

### pS2 and cathepsin D expression

Total RNA, transferred to a Hybond+ nylon membrane (Amersham International, Buckinghamshire, UK), was probed with double-stranded, biotin-labelled (non-radioactive Random Octamer Labelling System, Tropix, Bedford, MA, USA) pS2 cDNA and 52K-9 cDNA, corresponding to most of the coding sequence of pS2 and cathepsin D mRNA. All RNA samples were also probed for 36B4 mRNA, which was used as an internal control. Blots were revealed by a chemiluminescent method (Northern Chemiluminescent Detection System, Tropix), based on streptavidin–alkaline–phosphatase conjugate and a substrate (CSPD®), which, upon dephosphorylation, emits a light at 477 nm revealable by autoradiography on Hyperfilm MP (Amersham). Autoradiographs were densitometrically scanned using an LKB Ultrascan XL laser densitometer. Densitometric readings were normalized for 36B4 RNA content, and data were expressed as relative expression levels.

### Gel mobility shift assay

Complementary oligodeoxyribonucleotide strands containing a consensus ERE (GATCCAGGTCACAGTGACCTGGGCCCG-27 bp) were end-labelled with τ [$^{32}$P]-ATP (110 000 GBq mmol$^{-1}$) with the $T_4$ polynucleotide kinase (Amersham). DNA-binding reactions were carried out in buffer containing 6 ng radiolabelled ERE, 380 fmol of recombinant human ER (Boehringer, Mannheim, Germany), 20 mM HEPES (pH 7.9), 60 mM potassium chloride, 5 mM magnesium chloride, 2 mM dithiothreitol, 10% glycerol, 100 μg bovine serum albumin and the indicated concentrations of drugs in 20 μl total volume at 20°C for 20 min followed by 15 min additional incubation at 37°C. Thereafter, the protein–DNA complexes were separated on 4% native polyacrylamide gels in 90 mM Tris–borate buffer containing 2.5 mM EDTA, pH 8.3, at a constant current of 25 mA at room temperature.

### Data analysis

Each experimental point represents the mean of four determinations obtained by Latin Square in three separate experiments. Variations in treated samples were expressed with respect to the control. Differences between DNA content means were evaluated by Student's *t*-test.

### RESULTS

### Biological effects of genistein and quercetin

### *Effects of genistein and quercetin on proliferation of MCF-7 cells*

Figure 1 shows the effect of increasing concentrations of genistein and quercetin (ranging from 0.5 to 20 μM) on the growth of MCF-7 cells cultured for 6 days in medium containing 2% FCS. Genistein exerted a biphasic effect, stimulating growth (up to 120% of the control, $P < 0.01$) at concentrations of less than 5 μM and causing a dose-dependent inhibition at higher concentrations. Quercetin did not influence cell growth up to 2.5 μM and dramatically inhibited growth at higher concentrations. Noteworthy was the lower $IC_{50}$ value for quercetin (4.9 μM) than for genistein (10 μM).

### *Effect of genistein and quercetin on hormone- and growth factor-stimulated growth*

The effect of quercetin and genistein on the growth of stimulated MCF-7 cells was evaluated at 1 μM and 5 μM concentrations, which do not significantly alter cell growth of unstimulated cells (Figure 2). Experiments were carried out in serum-free medium. As already reported in our previous studies (Cappelletti et al, 1993), oestradiol and transforming growth factor α (TGF-α) caused a statistically significant ($P < 0.01$) stimulation of MCF-7 cell growth ranging from +50% to +20% respectively.

Quercetin and genistein efficiently and significantly ($P < 0.01$) counteracted the stimulation by oestradiol and TGF-α (Figure 2), which is known to mediate the oestrogenic stimulation of growth in the cell line.

*© 1999 Cancer Research Campaign* 

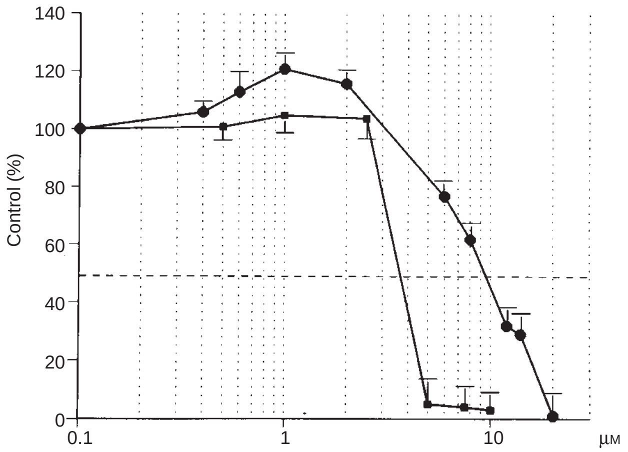

**Figure 1** Effect of various doses (0.5–20 μM) of genistein (●–●) and quercetin (■–■) on the growth of MCF-7. Cells were plated in 24-well culture dishes at a cell density of 15 000 cells per well and allowed to attach for 24 h. Thereafter, 2% FCS medium containing the substances to be tested was added and changed every 3 days. Experiments were stopped at day 7 when the cells were still in their exponential phase of growth. Each point is the average of three independent experiments performed in quadruplicate (Latin Square)

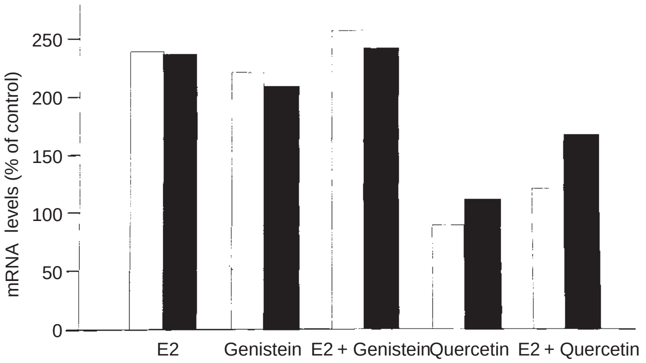

**Figure 3** Transcriptional regulation of cathepsin D and pS2 by genistein and quercetin alone or in combination with oestradiol. Total RNA was extracted by the Ultraspec-II RNA extraction system from MCF-7 cells treated for 48 h with 10 nM 17β-oestradiol, 5 μM genistein, or 1 μM quercetin in 2% DCC-FCS. RNA samples were run on 1% agarose formaldeyde-denaturing gel, blotted on a nylon membrane (Hybond+, Amersham) and probed with double-stranded cDNA probes pS2, 52K-9 and 36B4. Autoradiographs were densitometrically scanned to qualitatively evaluate pS2 and cathepsin D expression. The graph represents densitometric determinations of pS2 (open bars) and cathepsin D (closed bars) corrected for variation in total loaded RNA and expressed in arbitrary units

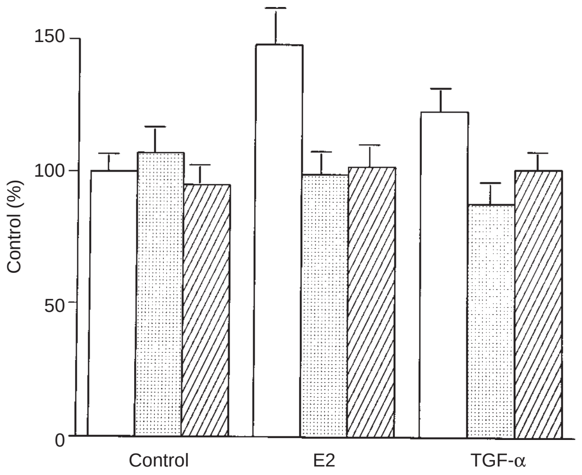

**Figure 2** Effect of quercetin and of genistein on the growth of MCF-7 cells treated with oestradiol and growth factors. Cells were plated in 24-well culture dishes at a cell density of 20 000 cells per well and allowed to attach for 24 h in complete growth medium. Thereafter, medium was replaced by $MOM_3$ medium containing the substances to be tested and was changed every 3 days. Experiments were stopped at day 7 when the cells were still in their exponential phase of growth. Hatched bars represent treatment with 1 μM quercetin, dotted bars represent treatment with 5 μM genistein, and open bars represent controls grown in the absence of any treatment or, when indicated, in the presence of 10 nM 17β-oestradiol or 1 ng $ml^{-1}$ TGF-α. Each bar is the mean of three separate experiments ± s.d.

### *Effect of genistein and quercetin on the expression of oestrogen-regulated genes*

We then addressed the ability of genistein and quercetin to inhibit oestradiol-promoted cell stimulation in an attempt to better understand the molecular basis for the anti-oestrogenic action of flavonoids on MCF-7 cell growth. The expression of ER and PR in cells treated with oestradiol alone or in combination with 5 μM genistein and 2.5 μM quercetin is reported in Table 1. Oestradiol caused a 42-fold induction of PR levels ($P < 0.001$), whereas genistein triggered a ninefold increase in PR expression ($P < 0.001$) and quercetin did not alter PR values. Induction of PR by oestradiol, and genistein, singly administered, was associated to a down-regulation of ER levels, which instead were not modified by treatment with quercetin. When the two phyto-oestrogens were combined with oestradiol, we observed an even stronger induction of PR (from 6 fmol $mgP^{-1}$ to 339 fmol $mgP^{-1}$) by genistein, whereas the combination of oestradiol and quercetin led to an attenuation of oestradiol-promoted PR induction (13.5-fold in the combined treatment ($P < 0.001$) versus 42-fold when cells were treated by oestradiol alone). At the 5 μM concentration, which abolished cell growth stimulation promoted by oestradiol, genistein induced a more than twofold stimulation of pS2 and cathepsin D transcription rate (Figure 3). Such stimulation was similar to that obtained by treatment with oestradiol (more than twofold), and the combined treatment resulted in a slightly stronger stimulation (not statistically different from that obtained with single-agent treatments).

Under the same experimental conditions, treatment with quercetin did not significantly influence the expression levels of pS2 and cathepsin D. When quercetin was combined with oestradiol, it almost completely counteracted the stimulation of pS2 promoted by oestradiol and caused a 50% reduction of oestradiol-induced cathepsin D stimulation (Figure 3).

### Molecular action of genistein and quercetin

### *Competition binding studies*

The ability of genistein and quercetin to compete for binding to ER sites under equilibrium conditions and in the presence of a saturating concentration of 16α-[$^{125}$I]-oestradiol was investigated over a range of competitor concentrations of 2.5 nM to 25 μM. Genistein competed with oestradiol for binding to the ER, with a lower relative affinity occupying as much as 80% of ER sites at the highest tested concentration (25 μM). In the concentration range used in our experiments, more than 70% of total receptor sites was occupied by genistein (Figure 4). In contrast, quercetin, tested over a similar range of concentrations, did not efficiently compete with

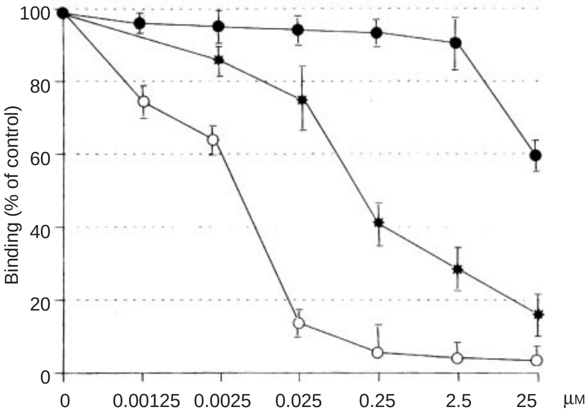

**Figure 4** Competition by genistein (●–●) and quercetin (*–*) for binding to cytoplasmic ER sites. Cytosol obtained from MCF-7 cells was incubated overnight at 0–4°C with 2.5 nM 16α-[[125]I]-oestradiol alone or in the presence of increasing amounts (1–10 000-fold molar excess) of 17β-oestradiol (○–○). Binding to the ER was assessed by DCC treatment and direct counting of protein-bound radioactivity oestradiol. It occupied less than 10% of ER sites when tested at 1–2.5 µM concentrations and only 40% of ER sites at the concentration 25 µM (Figure 4).

### *Effect of genistein and quercetin on steroid receptor metabolism*

MCF-7 cells grown in medium supplemented with 2% FCS were treated for 6 days with 1 µM quercetin or 5 µM genistein. At the end of the treatment, cells were harvested by trypsinization and processed for cytoplasmic and nuclear ER determination. Results are shown in Table 2. Genistein, and to a lesser extent quercetin, although unable to interact directly with the oestrogen ligand site, significantly ($P < 0.001$) down-regulated cytoplasmic ER levels, as expected for a true oestrogen agonist, and genistein also promoted a tighter association of the receptor with the nucleus.

### *Gel mobility shift assay*

To further clarify the molecular basis for the agonistic–antagonistic activity of genistein and to understand the mechanism of the antagonistic activity exerted by quercetin, we performed a gel mobility assay using purified ER and a labelled double-stranded ERE consensus sequence (Figure 5). Gel electrophoresis of samples containing the pure ER preincubated with $10^{-8}$ M oestradiol and the labelled oligonucleotide revealed the appearance of a retarded band corresponding to the ER–ERE complex since it was supershifted by the addition of a specific anti-ER antibody. A less intense band characterized by similar mobility was observed in control samples (without oestradiol). Pretreatment of the ER with genistein, in the presence or in the absence of oestradiol, induced the formation of a complex characterized by mobility identical to that obtained in the control and in the oestradiol-treated samples. The finding indicates that the DNA-binding properties of the oestradiol–ER complex and of the genistein–ER complex are indistinguishable and justifies the transcriptional induction of oestrogen-regulated genes by genistein. However, quercetin, although unable to compete with oestradiol in binding to the ER, also determined the formation of a retarded band electrophoretically indistinguishable from that observed with oestradiol and whose intensity appeared to be dose-dependent. Such a finding is therefore in agreement with the previous observation of a tighter binding of ER in the nucleus upon treatment of cells with quercetin.

### DISCUSSION

We compared the antiproliferative activity of genistein and quercetin in MCF-7, a typically hormone-sensitive breast cancer

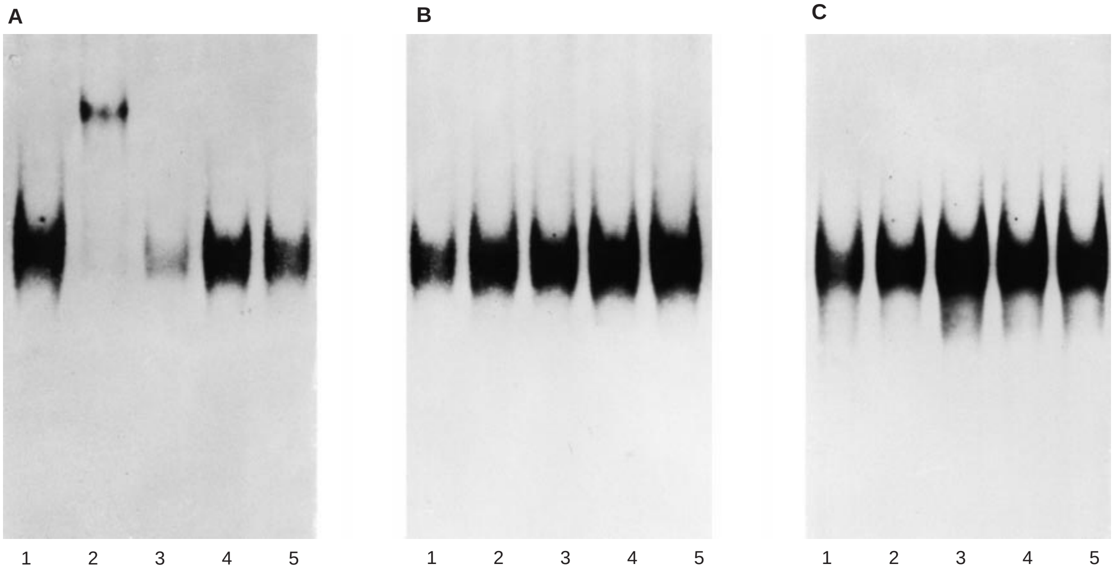

**Figure 5** Gel mobility assay of recombinant ER to the specific consensus sequence (GATCCAGGTCACAGTGACCTGGGCCCG-27-bp). Binding of radiolabelled ERE in the presence of: (**A**) $10^{-8}$ M oestradiol (lane 1), $10^{-8}$ M oestradiol and anti-ER antibody (HC-20, Santa Cruz, CA, USA) (lane 2), a 30-fold excess of unlabelled ERE (lane 3), $10^{-8}$ M oestradiol, and a 30-fold excess of unlabelled aspecific competitor SP1 (lane 4), control without ligand (lane 5); (**B**) control without oestradiol (lane 1), genistein 2.5 and 10 µm in the absence (lanes 2 and 3) and in the presence of $10^{-8}$ M oestradiol (lanes 4 and 5); (**C**) control without oestradiol (lane 1) and 1 and 10 µM quercetin in the absence (lanes 2 and 3) and in the presence of $10^{-8}$ M oestradiol (lanes 4 and 5)

*© 1999 Cancer Research Campaign*

cell line, in order to gain insight into their molecular mechanism of action at the ER level. Both phyto-oestrogens, when tested at concentrations that do not affect unstimulated cell growth, completely abolished stimulation promoted by oestradiol and by TGF-α, which is known to mediate oestradiol-promoted growth in such cell lines (Bates et al, 1986; Cappelletti et al, 1986). Based on our cell growth experiments, an anti-oestrogenic activity, at least on oestrogen- and TGF-α-mediated cell stimulation, was shown for both compounds. Such effects were observed at concentrations of genistein likely to be locally found in breast tissue of subjects with a high dietary intake of soy (Zava and Duwe, 1997).

In the case of quercetin, the antagonistic activity could also be evidenced by the expression of oestrogen-regulated genes. In fact, quercetin did not down-regulate cytoplasmic ER levels, as did oestradiol and genistein, and also did not increase PR expression, but it counteracted oestradiol-stimulated PR protein induction. The biocharacter of genistein and quercetin was also studied by investigating the expression of pS2 and cathepsin D genes at the RNA level. The findings on steroid receptor, pS2 and cathepsin D expression suggest that quercetin has an antagonistic potential not only on oestradiol-stimulated growth, but also on oestradiol-stimulated gene transcription. Therefore, based on the phenomenological data collected in our study, genistein could be defined as an agonistic–antagonist, depending on biological effect and concentration, whereas quercetin appears to behave like a pure oestrogen antagonist.

The study then addressed the molecular basis for such effects. Since a common step in the mechanism of action of anti-oestrogens is the specific high-affinity binding to the ER, we defined through competition studies the relative affinities of genistein and quercetin for ER. The ability of genistein to compete with oestradiol for binding to the ER could represent a necessary, but insufficient condition to exert an agonistic or an antagonistic effect, or, as frequently happens, a mixed agonistic–antagonistic activity. However, the lack of competition of quercetin for the oestradiol binding site prompted us to look for alternative antagonistic mechanisms. In fact, it could be hypothesized that the antagonistic activity is not mediated by a direct interaction with the ER binding site and may involve other domains of the ER protein, possibly leading to impairment of dimerization or a steric conformation with a weaker transcriptional activity.

The initiation of ER-regulated gene transcription requires a tight and specific interaction of the ER with its responsive element. The tightness of such an interaction is indirectly reflected by the so-called nuclear translocation process, whose practical consequence is recovery of the bulk of receptors in the nuclear (upon high salt extraction procedures) rather than in the soluble cytoplasmic fraction. In fact, the latter contains only those nuclear receptors loosely associated to the nucleus and therefore prone to leak into the cytoplasmic fraction during homogenization in hypotonic buffer.

We therefore investigated the subcellular localization of the ER after in vivo treatment with phyto-oestrogens. A tighter nuclear association of the ER was induced as expected by genistein, but surprisingly also by quercetin. We further investigated the specific interaction between the pure ER protein incubated in the presence of oestradiol and phyto-oestrogens with the specific radiolabelled ERE sequence. A specific interaction, as suggested by the retarded band observed in the gels and corresponding to the ER–ERE complex, was observed in control samples (as already described by Brown and Sharp, 1990), with an intensity that increased upon treatment with oestradiol, genistein or quercetin. Based on such data, we may conclude that genistein binds to the ER at the oestrogen binding site, and the formed complex interacts specifically with the ERE, thereby promoting the transcription of oestradiol-regulated genes. Quercetin, in contrast, does not bind the oestrogen binding site but probably interacts with some other sites: such interaction causes a conformational change in the ER protein, which leads to an increased binding to the ERE, but the formed ER–ERE complex is unable to activate transcription. The same type of interaction could also occur in the presence of oestradiol and determine a conformational change of the oestrogen-occupied receptor, which allows interaction with ER but impairs activation of gene transcription by oestrogens.

We are unable at present to better define the conformational change induced by quercetin on the free and occupied receptor protein because electrophoretic mobility of the retarded bands was indistinguishible. However, it may be hypothesized that the conformational variation induced by interaction with quercetin impairs the interaction between the ER–ERE complex and the co-activator and co-integrator proteins necessary for a productive contact with the basal transcription machinery. If such a hypothesis is true, molecules like the phyto-oestrogen quercetin may represent an interesting tool to better understand the interaction between ER and the numerous nuclear receptor co-activators and co-repressors recently described (Horwitz et al, 1996).

Of course, such an explanation about the molecular basis of the agonistic and antagonistic activity of flavonoids is very speculative and takes into account only ER-mediated effects of genistein and quercetin. Genistein and quercetin both have pleiotropic biological effects mainly due to their activity on enzymes of the signal transduction pathway (Singhal et al, 1995) and the enzymes of energy metabolism (Lang and Racker, 1974). Therefore, it may not be excluded that, depending on the tested dose, flavonoid-induced variations in multiple cellular processes may affect ER-regulated gene transcription and oestradiol-controlled cell proliferation.

### ACKNOWLEDGEMENTS

We thank B Johnston for editing the manuscript.

### REFERENCES

Adlercreutz H (1990) Western diet and western diseases: some hormonal and biochemical mechanisms and associations. *Scand J Clin Lab Invest* **50**: 3–23

Adlercreutz H, Godin BR, Gorbach SL, Hockerstedt KAV, Watanabe S, et al (1995) Soybean phytoestrogen intake and cancer risk. *J Nutr* **125**: 757S–770S

Akiyama T, Ishida J, Nikagawa H, Watanabe S, Itoh N, Shibuy AM and Fukamo Y (1987) Genistein, a specific inhibitor of tyrosine-specific protein kinases. *J Biol Chem* **262**: 5592–5595

Barnes S, Grubbs C, Setchell KDR and Carlson J (1990) Soybeans inhibit mammary tumors in models of breast cancer. In: *Mutagens and Carcinogens in the Diet,* Pariza M (ed), pp. 239–253. Wiley-Liss: New York

Barnes S (1995) Effect of genistein on in vitro and in vivo models of cancer. *J Nutr* **125**: 777S–783S

Bates SE, McManaway ME, Lippman ME and Dickson RB (1986) Characterization of estrogen responsive transforming activity in human breast cancer cells. *Cancer Res* **46**: 1707–1713

Brown M and Sharp PA (1990) Human estrogen receptor forms multiple protein–DNA complexes. *J Biol Chem* **265**: 11238–11243

Burton KH (1956) A study of the conditions and mechanism of diphenylamine reaction for colorimetric determination of deoxyribonucleic acid. *Biochem J* **62**: 315–323

*© 1999 Cancer Research Campaign*

Cappelletti V, Brivio M, Miodini P, Granata G, Coradini D and Di Fronzo G (1988) Simultaneous estimation of epidermal growth factor receptors and steroid receptors in a series of 136 resectable primary breast tumors. *Tumor Biol* **9**: 200–211

Cappelletti V, Ruedl C, Miodini P, Fioravanti L, Coradini D, Di Fronzo G et al (1993) Paracrine interactions in co-culture of hormone-dependent and independent breast cancer cells. *Breast Cancer Res Treat* **26**: 275–281

Cappelletti V, Miodini P, Fioravanti L and Di Fronzo G (1995) Effect of progestin treatment on estradiol- and growth factor-stimulated breast cancer cell lines. *Anticancer Res* **15**: 2551–2556

Fioravanti L, Cappelletti V, Miodini P, Ronchi E, Brivio M and Di Fronzo G (1998) Genistein in the control of breast cancer cell growth: insights into the mechanism of action in vitro. *Cancer Lett* **130**: 143–152

Horwitz KB, Jackson TA, Brain DL, Richer JK, Takimoto GS and Tung L (1996) Nuclear receptor coactivators and corepressors. *Mol Endocrinol* **10**: 1167–1177

Katzenellenbogen JA, O'Malley BW and Katzenellenbogen BS (1996) Tripartite steroid hormone receptor pharmacology: interaction with multiple effector sites as a basis for the cell-and-promoter specific action of these hormones. *Mol Endocrinol* **10**: 119–131

Kühnau J (1976) The flavonoids, a class of semi-essential food components: their role in human nutrition. *Wld Rev Nutr Diet* **24**: 117–191

Lamartiniere CA, Moore JB, Brown NM, Thompson R, Hardin MJ and Barnes S (1995) Genistein suppresses mammary cancer in rats. *Carcinogenesis* **16**: 2833–2840

Lang DR and Racker E (1974) Effect of quercetin and F1 inhibition on mitochondrial ATPase and energy-linked reactions in sub-mitochondrial particles. *Biochem Biophys Acta* **333**: 180–186

Larocca LM, Piantelli M, Leone G, Sica S, Teofili L, Benedetti Panici P, Scambia G, Mancuso S, Capelli A and Rancelletti FO (1990) Type-II oestrogen binding sites in acute lymphoid and myeloid leukemias: growth inhibitory effect of oestrogen and flovonoids. *Br J Haematol* **75**: 489–495

Lock M (1991) Contested meanings of the menopause. *Lancet* **337**: 1270–1272

Markovits J, Linassier C, Fosse P, Couprie J, Pierre J, Jacquemin-Sablon et al (1989) Inhibitory effects of the tyrosine kinase inhibitor genistein on mammalian DNA polymerase II. *Cancer Res* **49**: 5111–5117

Martin PM, Horwitz KB, Ryan DS and McGuire WL (1978) Phytoestrogen interaction with estrogen receptors in human breast cancer cells. *Endocrinology* **103**: 1860–1867

Ronchi E, Granata G, Brivio M, Coradini D, Miodini P and Di Fronzo G (1986) A double-labelling assay for simultaneous estimation and characterization of estrogen and progesterone receptors using radioiodinated estradiol and tritiated Org2058. *Tumori* **72**: 251–257

Ross PD, Norimatsu H, Davis JW and Yano K (1991) A comparison of hip fracture incidence among native Japanese, Japanese Americans and American Caucasians. *Am J Epidemiol* **133**: 801–809

Scambia G, Rannelletti FO, Benedetti Panici P, Piantelli M, Bonanno G, De Vincenzo R, Ferrandina G, Rumi C, Larocca LM and Mancuso S (1990) Inhibitory effect of quercetin on OVCA 433 cells and presence of type-II oestrogen binding sites in primary ovarian tumors and cultured cells. *Br J Cancer* **62**: 942–946

Scambia G, Ranelletti FO, Benedetti-Panici P, Piantelli M, De Vincenzo R, Ferrandina G, Bonanno G, Capelli A and Mancuso S (1993) Quercetin induces type-II estrogen binding sites in estrogen-receptor-negative (MDAMB-231) and estrogen-receptor-positive (MCF-7) human breast cancer cell lines. *Int J Cancer* **54**: 462–466

Setchell KDR and Adlercreutz H (1988) Mammalian lignans and phytoestrogens. Recent studies on their formation, metabolism and biological role in health and disease. In: *Role of the Gut Flora in Toxicity and Cancer*, Rowland IR (ed), pp. 315–345. Academic Press: London

Shiu-Ming Kuo (1996) Antiproliferative potency of structurally distinct dietary flavonoids on human colon cancer cells. *Cancer Lett* **110**: 41–48

Singhal RL, Yeah YA, Prajda N, Olah E, Sledge GS and Weber G (1995) Quercetin down-regulates signal transduction in human breast carcinoma cells. *Biochem Biophys Res CommUN* **208**: 425–431

Soto AM and Sonnenschein C (1985) The role of estrogen on the proliferation of human breast tumor cells (MCF-7). *J Steroid Biochem* **32**: 87–94

Yamagihara K, Ito A, Toge T and Numoto M (1993) Antiproliferative effects of isoflavones on human cancer cell lines established from the gastrointestinal tract. *Cancer Res* **53**: 5815–5821

Zava DT and Duwe G (1997) Estrogenic and antiproliferative properties of genistein and other fLavonoids in human breast cancer cells in vitro. *Nutr Cancer* **27**: 31–40

*© 1999 Cancer Research Campaign*

## References

[PDF_00495] Adlercreutz C. H., Goldin B. R., Gorbach S. L., Höckerstedt K. A., Watanabe S., Hämäläinen E. K., Markkanen M. H., Mäkelä T. H., Wähälä K. T., Adlercreutz T. (1995). Soybean phytoestrogen intake and cancer risk.. J Nutr.

[PDF_00493] Adlercreutz H. (1990). Western diet and Western diseases: some hormonal and biochemical mechanisms and associations.. Scand J Clin Lab Invest Suppl.

[PDF_00497] Akiyama T., Ishida J., Nakagawa S., Ogawara H., Watanabe S., Itoh N., Shibuya M., Fukami Y. (1987). Genistein, a specific inhibitor of tyrosine-specific protein kinases.. J Biol Chem.

[PDF_00510] BURTON K. (1956). A study of the conditions and mechanism of the diphenylamine reaction for the colorimetric estimation of deoxyribonucleic acid.. Biochem J.

[PDF_00503] Barnes S. (1995). Effect of genistein on in vitro and in vivo models of cancer.. J Nutr.

[PDF_00500] Barnes S., Grubbs C., Setchell K. D., Carlson J. (1990). Soybeans inhibit mammary tumors in models of breast cancer.. Prog Clin Biol Res.

[PDF_00508] Brown M., Sharp P. A. (1990). Human estrogen receptor forms multiple protein-DNA complexes.. J Biol Chem.

[PDF_00517] Cappelletti V., Brivio M., Miodini P., Granata G., Coradini D., Di Fronzo G. (1988). Simultaneous estimation of epidermal growth factor receptors and steroid receptors in a series of 136 resectable primary breast tumors.. Tumour Biol.

[PDF_00524] Cappelletti V., Miodini P., Fioravanti L., Di Fronzo G. (1995). Effect of progestin treatment on estradiol-and growth factor-stimulated breast cancer cell lines.. Anticancer Res.

[PDF_00521] Cappelletti V., Ruedl C., Miodini P., Fioravanti L., Coradini D., Di Fronzo G., Silvestrini R. (1993). Paracrine interaction in co-culture of hormone-dependent and independent breast cancer cells.. Breast Cancer Res Treat.

[PDF_00505] Dickson R. B., Bates S. E., McManaway M. E., Lippman M. E. (1986). Characterization of estrogen responsive transforming activity in human breast cancer cell lines.. Cancer Res.

[PDF_00527] Fioravanti L., Cappelletti V., Miodini P., Ronchi E., Brivio M., Di Fronzo G. (1998). Genistein in the control of breast cancer cell growth: insights into the mechanism of action in vitro.. Cancer Lett.

[PDF_00530] Horwitz K. B., Jackson T. A., Bain D. L., Richer J. K., Takimoto G. S., Tung L. (1996). Nuclear receptor coactivators and corepressors.. Mol Endocrinol.

[PDF_00532] Katzenellenbogen J. A., O'Malley B. W., Katzenellenbogen B. S. (1996). Tripartite steroid hormone receptor pharmacology: interaction with multiple effector sites as a basis for the cell- and promoter-specific action of these hormones.. Mol Endocrinol.

[PDF_00576] Kuo S. M. (1996). Antiproliferative potency of structurally distinct dietary flavonoids on human colon cancer cells.. Cancer Lett.

[PDF_00536] Kühnau J. (1976). The flavonoids. A class of semi-essential food components: their role in human nutrition.. World Rev Nutr Diet.

[PDF_00538] Lamartiniere C. A., Moore J. B., Brown N. M., Thompson R., Hardin M. J., Barnes S. (1995). Genistein suppresses mammary cancer in rats.. Carcinogenesis.

[PDF_00544] Larocca L. M., Piantelli M., Leone G., Sica S., Teofili L., Panici P. B., Scambia G., Mancuso S., Capelli A., Ranelletti F. O. (1990). Type II oestrogen binding sites in acute lymphoid and myeloid leukaemias: growth inhibitory effect of oestrogen and flavonoids.. Br J Haematol.

[PDF_00548] Lock M. (1991). Contested meanings of the menopause.. Lancet.

[PDF_00549] Markovits J., Linassier C., Fossé P., Couprie J., Pierre J., Jacquemin-Sablon A., Saucier J. M., Le Pecq J. B., Larsen A. K. (1989). Inhibitory effects of the tyrosine kinase inhibitor genistein on mammalian DNA topoisomerase II.. Cancer Res.

[PDF_00552] Martin P. M., Horwitz K. B., Ryan D. S., McGuire W. L. (1978). Phytoestrogen interaction with estrogen receptors in human breast cancer cells.. Endocrinology.

[PDF_00555] Ronchi E., Granata G., Brivio M., Coradini D., Miodini P., Di Fronzo G. (1986). A double-labeling assay for simultaneous estimation and characterization of estrogen and progesterone receptors using radioiodinated estradiol and tritiated Org 2058.. Tumori.

[PDF_00559] Ross P. D., Norimatsu H., Davis J. W., Yano K., Wasnich R. D., Fujiwara S., Hosoda Y., Melton L. J. (1991). A comparison of hip fracture incidence among native Japanese, Japanese Americans, and American Caucasians.. Am J Epidemiol.

[PDF_00562] Scambia G., Ranelletti F. O., Panici P. B., Piantelli M., Bonanno G., De Vincenzo R., Ferrandina G., Rumi C., Larocca L. M., Mancuso S. (1990). Inhibitory effect of quercetin on OVCA 433 cells and presence of type II oestrogen binding sites in primary ovarian tumours and cultured cells.. Br J Cancer.

[PDF_00567] Scambia G., Ranelletti F. O., Panici P. B., Piantelli M., De Vincenzo R., Ferrandina G., Bonanno G., Capelli A., Mancuso S. (1993). Quercetin induces type-II estrogen-binding sites in estrogen-receptor-negative (MDA-MB231) and estrogen-receptor-positive (MCF-7) human breast-cancer cell lines.. Int J Cancer.

[PDF_00578] Singhal R. L., Yeh Y. A., Praja N., Olah E., Sledge G. W., Weber G. (1995). Quercetin down-regulates signal transduction in human breast carcinoma cells.. Biochem Biophys Res Commun.

[PDF_00581] Soto A. M., Sonnenschein C. (1985). The role of estrogens on the proliferation of human breast tumor cells (MCF-7).. J Steroid Biochem.

[PDF_00583] Yanagihara K., Ito A., Toge T., Numoto M. (1993). Antiproliferative effects of isoflavones on human cancer cell lines established from the gastrointestinal tract.. Cancer Res.

[PDF_00586] Zava D. T., Duwe G. (1997). Estrogenic and antiproliferative properties of genistein and other flavonoids in human breast cancer cells in vitro.. Nutr Cancer.

